# Histological phenotypic subtypes predict recurrence risk and response to adjuvant chemotherapy in patients with stage III colorectal cancer

**DOI:** 10.1002/cjp2.171

**Published:** 2020-05-13

**Authors:** Antonia K Roseweir, James H Park, Sanne ten Hoorn, Arfon GMT Powell, Susan Aherne, Campbell SD Roxburgh, Donald C McMillan, Paul G Horgan, Elizabeth Ryan, Kieran Sheahan, Louis Vermeulen, James Paul, Andrea Harkin, Janet Graham, Owen Sansom, David N Church, Ian Tomlinson, Mark Saunders, Tim J Iveson, Joanne Edwards

**Affiliations:** ^1^ School of Medicine University of Glasgow Glasgow UK; ^2^ Institute of Cancer Sciences University of Glasgow Glasgow UK; ^3^ Laboratory for Experimental Oncology and Radiobiology, Center for Experimental Molecular Medicine, Amsterdam UMC University of Amsterdam, Cancer Center Amsterdam, Oncode Institute Amsterdam The Netherlands; ^4^ Division of Cancer and Genetics Cardiff University Cardiff UK; ^5^ School of Medicine University College Dublin and Centre for Colorectal Disease, St Vincent's University Hospital Dublin Ireland; ^6^ CRUK Clinical Trials Unit The Beatson West of Scotland Cancer Centre, Gartnavel Hospital Glasgow UK; ^7^ Wellcome Centre for Human Genetics University of Oxford Oxford UK; ^8^ NIHR Oxford Biomedical Research Centre Oxford University Hospitals NHS Foundation Trust, John Radcliffe Hospital Oxford UK; ^9^ Edinburgh Cancer Research Centre, IGMM University of Edinburgh Edinburgh UK; ^10^ The Christie NHS Foundation Trust Manchester UK; ^11^ Southampton University Hospital NHS Foundation Trust Southampton UK

**Keywords:** colorectal cancer, subtyping, histopathology, adjuvant treatment, recurrence, precision medicine

## Abstract

Histological ‘phenotypic subtypes’ that classify patients into four groups (immune, canonical, latent and stromal) have previously been demonstrated to stratify survival in a stage I–III colorectal cancer (CRC) pilot cohort. However, clinical utility has not yet been validated. Therefore, this study assessed prognostic value of these subtypes in additional patient cohorts along with associations with risk of recurrence and response to chemotherapy. Two independent stage I–III CRC patient cohorts (internal and external cohort) were utilised to investigate phenotypic subtypes. The primary endpoint was disease‐free survival (DFS) and the secondary endpoint was recurrence risk (RR). Stage II–III patients, from the SCOT adjuvant chemotherapy trial, were utilised to further validate prognostic value and for exploratory analysis assessing associations with adjuvant chemotherapy. In an 893‐patient internal cohort, phenotypic subtype independently associated with DFS (*p* = 0.025) and this was attenuated in stage III patients (*p* = 0.020). Phenotypic subtype also independently associated with RR (*p* < 0.001) in these patients. In a 146‐patient external cohort, phenotypic subtype independently stratified patients by DFS (*p* = 0.028), validating their prognostic value. In 1343 SCOT trial patients, the effect of treatment type significantly depended on phenotypic subtype (*p*_interaction_ = 0.011). Phenotypic subtype independently associated with DFS in stage III patients receiving FOLFOX (*p* = 0.028). Furthermore, the immune subtype significantly associated with better response to FOLFOX compared to CAPOX adjuvant chemotherapy in stage III patients (*p* = 0.013). In conclusion, histological phenotypic subtypes are an effective prognostic classification in patients with stage III CRC that associates with risk of recurrence and response to FOLFOX adjuvant chemotherapy.

## Introduction

There has been an enormous effort to develop a prognostic classification of colorectal cancer (CRC) based mainly on transcriptomic analysis of tumours [1, 2]. We recently proposed a histological phenotypic subtyping method based on phenotypic characteristics of the tumour microenvironment (immune and stromal infiltrate) and tumour proliferation [3], extrapolated from the consensus molecular subtypes (CMS), that could translate more readily to the clinic [1]. This method classifies into four prognostic groups – immune, canonical, latent and stromal – in a discovery cohort of 237 patients with stage I–III CRC [3], but requires validation.

Establishing distinct patient groups will allow development of personalised treatment approaches for CRC, as evidenced by the use of mismatch repair deficiency for response to immunotherapy [4]. There is currently a lack of biomarkers to predict response to adjuvant chemotherapy, particularly important for CRC and 5‐FU‐based adjuvant chemotherapy, as the SCOT trial recently demonstrated that patients receiving CAPOX (capecitabine and oxaliplatin) have similar survival with 3‐ versus 6‐months duration, whereas patients receiving FOLFOX (bolus and infused fluorouracil with oxaliplatin) require 6‐months duration [5]. This highlights the importance of identifying which patients need to receive the longer more invasive FOLFOX regimen compared to the potentially shorter less invasive CAPOX regimen. Histological phenotypic subtyping could provide such a tool.

Hence, the primary aim of this study was to validate the prognostic value of the histological phenotypic subtypes in distinct stage I–III CRC internal and external cohorts. The secondary aim was to assess associations with risk of recurrence. Finally, the exploratory aim was to investigate adjuvant chemotherapy in a subset of stage II–III CRC patients from the SCOT trial to assess associations with treatment type or duration.

## Methods

### Study cohorts

In the internal cohort there were 1030 patients who had undergone a potentially curative resection for stage I–III CRC between 1997 and 2007 at the Glasgow Royal Infirmary, Western Infirmary or Stobhill Hospitals (Glasgow, UK, GSH/18/ON/007; Ethics No. 16/WS/0207). In the external validation cohort, there were 166 patients who had undergone a potentially curative resection for stage I–III CRC between 1992 and 2016 at St Vincent's University Hospital (Dublin, Ireland) or the Academic Medical Center (Amsterdam, The Netherlands, AMC‐AJCCII‐90; Ethics No. W12/011/12.17.0020). In the TransSCOT cohort there was tissue available from 3000 patients, derived from the SCOT adjuvant chemotherapy trial (ISRCTN no. 59757862; 6088 patients) who had undergone potentially curative resection for high‐risk stage II or stage III CRC between 2008 and 2013 within the UK.

**Figure 1:**
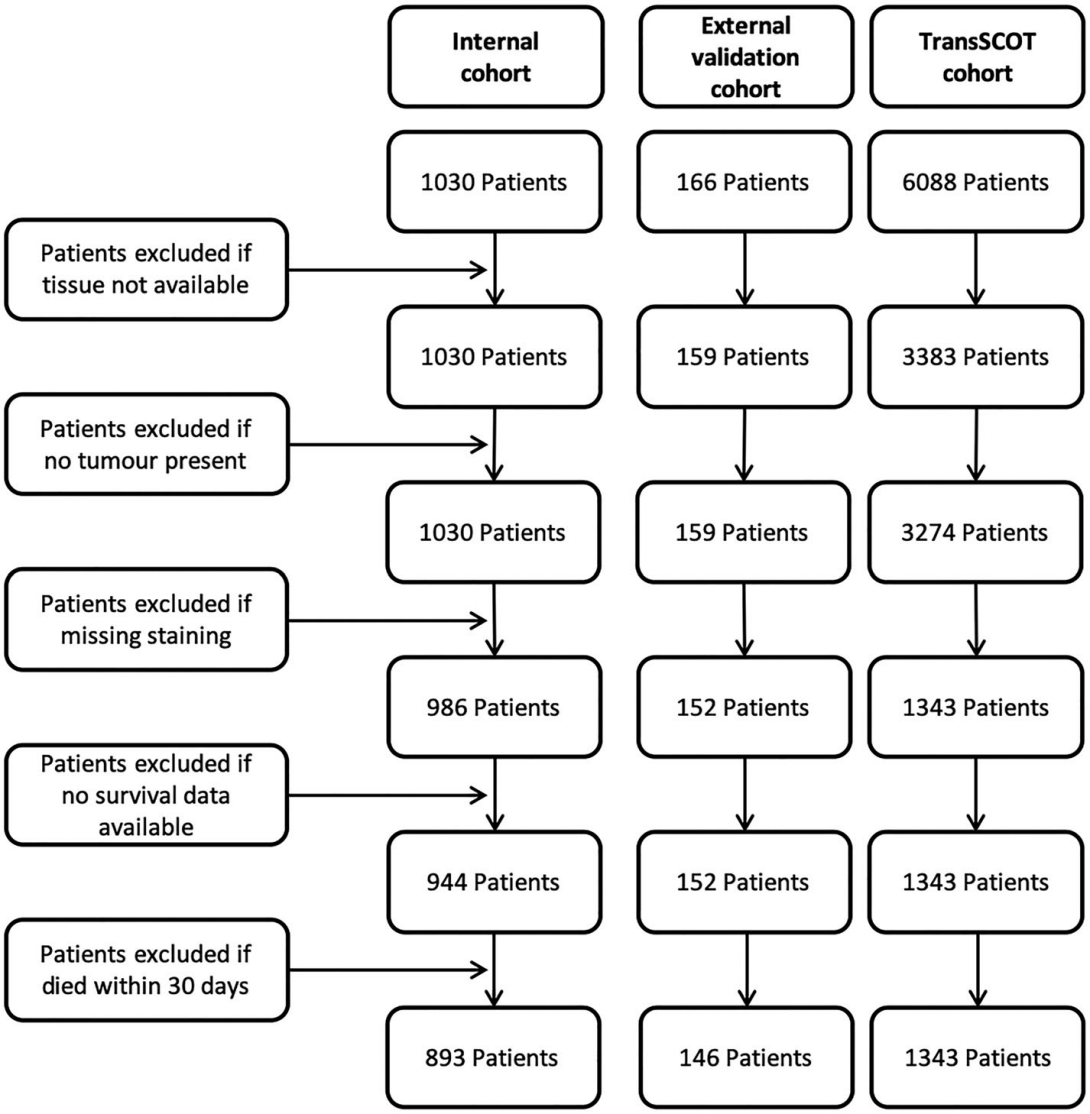
Consort diagram for the patient cohorts. Flow diagram showing criteria for exclusion of patients from the study comparing the three cohorts; internal cohort, external validation cohort and TransSCOT cohort.

All patients were followed up for at least 3 years and patients who died within 30 days of surgery, had no tissue/survival data available or for whom staining missing were excluded from the analysis, providing 893 eligible patients within the internal cohort, 146 eligible patients within the external validation cohort and 1343 eligible patients within the TransSCOT cohort (Figure 1). The study complied with the Declaration of Helsinki and individual ethics committee guidelines. It was not appropriate to involve patients or the public in the design, conduct, reporting or dissemination of this research.

### Patient cohort clinicopathological characteristics

Tumours were staged using the fifth edition (UK) or seventh edition (Amsterdam and Dublin) of the AJCC/UICC‐TNM staging system. In the internal cohort, the presence of venous invasion was assessed using elastica staining. Tumour differentiation was graded as well/moderate or poor in accordance with Royal College of Pathologists guidelines [6] and additional data were taken from pathology reports. Mismatch repair status was available for the internal and external validation cohorts. Following surgery, patients with stage III or high‐risk stage II disease and without significant co‐morbid disease precluding adjuvant treatment were considered for 5‐fluorouracil‐based chemotherapy. For the TransSCOT cohort, all patients were treated with FOLFOX (bolus and infused fluorouracil with oxaliplatin) or CAPOX (capecitabine and oxaliplatin) adjuvant chemotherapy randomised to 3‐ or 6‐months duration. Date and site of recurrence and cause of death were crosschecked using electronic case records.

### Assessment of phenotypic subtypes

Phenotypic subtype measures were assessed on full sections taken from the deepest point of invasion. For the internal, Amsterdam and TransSCOT cohorts, slides were assessed by a single observer (AKR or JHP) with 10% co‐scored by the other observer; interclass correlation coefficient was >0.7 for all markers. The classification was externally validated by an independent observer (SA) at St Vincent's Hospital, Dublin, with 10% co‐scored by a second observer (AKR); interclass correlation coefficient was >0.7 for all markers. Local inflammatory cell infiltrate was visually assessed using the Klintrup–Makinen (KM) grade [7]. Briefly, the KM grade was assessed on a H&E section at the invasive margin; a score of 0–1 (no increase or mild/patchy increase in inflammatory cells) was graded as weak and a score of 2–3 (prominent inflammatory reaction forming a band at the invasive margin, or florid cup‐like infiltrate at the invasive edge with destruction of cancer cell islands) was graded as strong. Stromal invasion was visually assessed using tumour stroma percentage (TSP) on an H&E section with a cut‐off value of 50% for low (≤50% stroma present) and high (>50% stroma present) [8]. Proliferation rate was assessed using Ki67 proliferation index with automated hotspot cell counts within a single pre‐determined field of view at ×400 magnification utilising the SlidePath digital image hub (Leica, UK). Proliferation rate cut‐offs were assessed by receiver operating characteristic (ROC) analysis in the internal cohort and 30% deemed to be the optimal cut‐off. Proliferation was graded as low (≤30%) or high (>30%).

### Study endpoints

The primary endpoint was disease‐free survival (DFS; measured from date of surgery/randomisation to date of recurrence at any location or death from any cause). The secondary endpoint was recurrence risk (RR: measured from date of surgery/randomisation to date of recurrence at any location or death from CRC). The exploratory endpoint was associations with adjuvant chemotherapy type and duration.

### Statistical analysis

The prospectively powered outcome analysis compared the immune (37%) and stromal (19%) subtypes. By using a two‐sided *α* = 0.05 analysis and assuming a hazard ratio (HR) of 2.0 and an immune subtype prevalence of 37%, a sample size of >225 patients gave >90% power to detect a survival difference between the immune and stromal subtypes. SPSS (version 25; IBM, New York, NY, USA) was used for statistical analysis. Kaplan–Meier and log‐rank analysis compared DFS or RR (adjusted for T‐stage, N‐stage and treatment duration when assessing chemotherapy interactions). The log‐rank for overall trend was reported for all DFS and RR survival analysis. HRs and CIs were calculated from univariate Cox regression survival analysis. Multivariable Cox regression survival analysis using a backward conditional elimination model and a statistical significance threshold of *P* value less than 0.1 was performed to identify independent prognostic biomarkers. A Cox proportional hazards interaction model was performed to assess interactions between phenotypic subtype and treatment type/duration. The study conformed to the REMARK guidelines [9] and statistical significance was set at *P* value less than 0.05. All statistical tests were two‐sided.

## Results

The internal cohort (*n* = 893) was utilised to validate the prognostic utility of the phenotypic subtypes in stage I–III CRC patients (for cohort characteristics, see supplementary material, Table S1). No adjuvant therapy data was available. Median follow‐up was 11.3 years (range 6.2–16.0 years) with 256 cancer deaths, 287 non‐cancer deaths and a 33% recurrence rate. 305 (34%) patients had an immune subtype, 248 (28%) a canonical subtype, 186 (21%) a latent subtype and 154 (17%) a stromal subtype. The immune, canonical and latent subtypes contained older patients with earlier stage cancer, however, the stromal subtype contained younger patients with later stage cancer (see supplementary material, Figure S1).

To ensure the hierarchy of the phenotypic subtype classification developed in the pilot study was robust, the three markers utilised were entered into multivariate analysis (see supplementary material, Table S2, *n* = 881) with KM grade (*p* = 0.002) and TSP (*p* = 0.073) but not Ki67 (*p* = 0.971) demonstrated to be independently prognostic for DFS. The hierarchy of the three markers was KM grade being considered first as it was independently prognostic for DFS, then TSP as it was trending towards being independently prognostic for DFS and finally Ki67 as it showed dependence on the other two markers. Hence, phenotypic subtype was defined as follows for all cohorts (Table 1): Immune – High KM‐grade, any TSP and any Ki67; stromal – low KM grade, high TSP and any Ki67; canonical – low KM‐grade, low TSP and high Ki67; and latent – low for all three markers.

**Table 1:** Phenotypic subtype classification.

	Immune	Canonical	Latent	Stromal
Intra‐tumour immune infiltrate (KM grade; 0–1/2–3)	High	Low	Low	Low
Stromal invasion (TSP; ≤50%/>50%)	Any	Low	Low	High
Proliferation rate (Ki67; ≤30%/>30%)	Any	High	Low	Any

**Figure 2:**
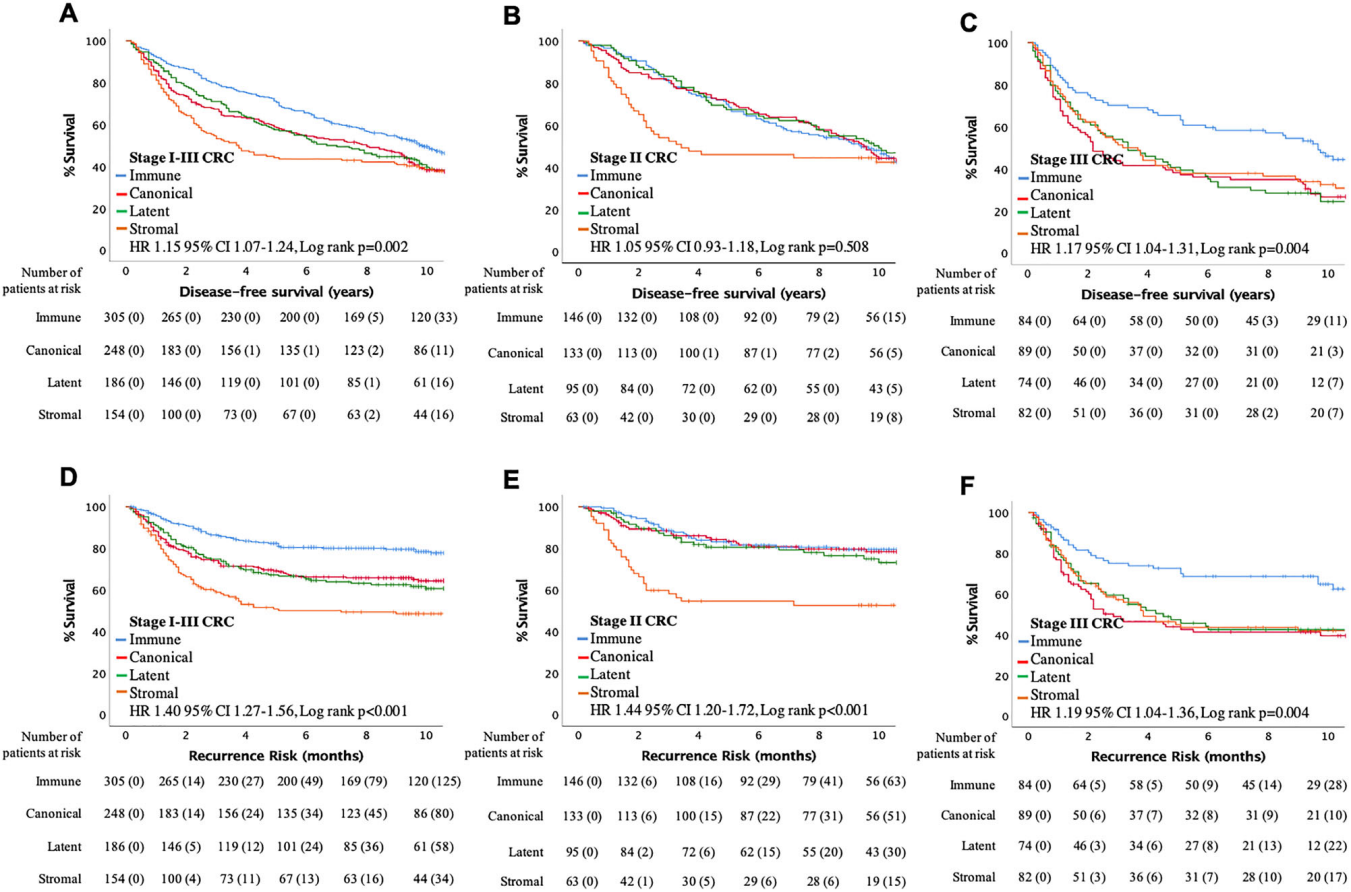
Phenotypic subtype stratifies survival and recurrence risk in stage I–III CRC patients within the internal cohort. (A–C)Kaplan–Meier curves showing association between phenotypic subtypes and disease‐free survival in patients with (A) stage I–III CRC (*n* = 893), (B) stage II CRC (*n* = 437) and (C) stage III CRC (*n* = 329). (D–F)Kaplan–Meier curves showing association between phenotypic subtypes and recurrence risk in patients with (D) stage I–III CRC (*n* = 893), (E) stage II CRC (*n* = 437) and (F) stage III CRC (*n* = 329).

To address the primary endpoint, associations with DFS were assessed (Figure 2A–C). Phenotypic subtype was shown to significantly stratify DFS (HR 1.15 95% CI 1.07–1.24, *p* = 0.002; Figure 2A), with the immune subtype having the best outcome and the stromal subtype the worst outcome. To investigate the phenotypic subtypes in important disease stages, patients were stratified into stage II or stage III disease. Phenotypic subtype did not stratify DFS in stage II CRC (HR 1.05 95% CI 0.93–1.18; Figure 2B); however, they did significantly associate with DFS in stage III CRC, with the immune subtype having significantly improved survival (HR 1.17 95% CI 1.04–1.31, *p* = 0.004; Figure 2C). Next to address the secondary endpoint, associations with RR were assessed (Figure 2D–F). Phenotypic subtype significantly associated with RR, with the immune subtype having a low risk and the stromal subtype having the highest risk (HR 1.40 95% CI 1.27–1.56, *p* < 0.001; Figure 1D). When assessing important disease stages, phenotypic subtype associated with RR in both stage II (HR 1.44 95% CI 1.20–1.72, *p* < 0.001; Figure 2E) and stage III (HR 1.19 95% CI 1.04–1.36, *p* = 0.004; Figure 2F) CRC. In stage II disease, only patients with a stromal subtype had a high risk of recurrence whereas, in stage III disease, canonical, latent and stromal patients were all at risk of recurrence.

To assess the phenotypic subtypes as an independent prognostic factor, the relationship between phenotypic subtype, common clinical factors and DFS or RR was examined in multivariate analysis (Table 2). For DFS in the full cohort (*n* = 893), age (*p* < 0.001), N‐stage (*p* < 0.001), vascular invasion (*p* = 0.008), peritoneal involvement (*p* < 0.001), tumour necrosis (*p* = 0.025) and phenotypic subtype (*p* = 0.025) were independently prognostic. In stage II disease (*n* = 437), only age (*p* < 0.001) remained independently prognostic. However, in stage III disease (*n* = 329), age (*p* = 0.004), T‐stage (*p* = 0.020), differentiation (*p* = 0.016), vascular invasion (*p* = 0.026), tumour perforation (*p* < 0.001) and phenotypic subtype (*p* = 0.020) remained independently prognostic. Therefore, phenotypic subtype is only an independent prognostic factor in stage III disease for DFS. For RR in the full cohort, N‐stage (*p* < 0.001), vascular invasion (*p* = 0.002), peritoneal involvement (*p* < 0.001), tumour perforation (*p* = 0.004) and phenotypic subtype (*p* < 0.001) were independent risk factors for recurrence. In stage II disease, only peritoneal involvement (*p* = 0.008) and phenotypic subtype (*p* < 0.001) remained independent risk factors for recurrence. Whereas in stage III disease, differentiation (*p* = 0.010), vascular invasion (*p* = 0.012), peritoneal involvement (*p* < 0.001), tumour perforation (*p* = 0.001) and phenotypic subtype (*p* = 0.011) remained independent risk factors for recurrence. Therefore, phenotypic subtype is a risk factor for recurrence in all patients with stage I–III CRC.

**Table 2:** Multivariate analysis of phenotypic subtypes, clinical factors and disease‐free survival in the internal cohort (*n* = 893).

	All patients (*n* = 889)				Stage II: patients (*n* = 437)				Stage III: patients (*n* = 329)			
	Univariate analysis HR (95% CI)	*P* value	Multivariate analysis HR (95% CI)	*P* value	Univariate analysis HR (95% CI)	*P* value	Multivariate analysis HR (95% CI)	*P* value	Univariate analysis HR (95% CI)	*P* value	Multivariate analysis HR (95% CI)	*P* value
Disease‐free survival												
Age												
<65/<65	1.69 (1.39–2.05)	<0.001	1.82 (1.50–2.22)	<0.001	1.89 (1.41–2.52)	<0.001	1.89 (1.41–2.52)	<0.001	1.42 (1.07–1.88)	0.015	1.54 (1.15–2.07)	0.004
T‐Stage												
1	–	<0.001	–	0.198	–	–	–	–	–	<0.001	–	0.02
2	1.33 (0.79–2.25)	0.285	1.48 (0.86–2.54)	0.158					5.81 (0.76–44.17)	0.089	5.09 (0.66–39.25)	0.119
3	1.68 (1.05–2.71)	0.032	1.44 (0.88–2.38)	0.151					5.64 (0.79–40.39)	0.085	3.86 (0.53–32–26)	0.184
4	2.37 (1.46–3.86)	<0.001	0.86 (0.40–1.87)	0.707					10.27 (1.43–73–68)	0.021	5.81 (0.79–42.85)	0.084
N‐stage												
0	–	<0.001	–	<0.001	–	–	–	–	–	0.189	–	–
1	1.58 (1.31–1.90)	<0.001	1.49 (1.23–1.81)	<0.001					3.27 (0.46–23.35)	0.238		
2	1.91 (1.46–2.48)	<0.001	1.68 (1.27–2.21)	<0.001					3.97 (0.55–28.61)	0.171		
Differentiation												
Poor/well or moderate	2.11 (1.51–2.96)	<0.001	1.29 (0.99–1.68)	0.057	1.22 (0.86–1.84)	0.349	–	–	1.46 (1.04–2.05)	0.028	1.54 (1.09–2.19)	0.016
Vascular Invasion												
No/yes	1.48 (1.24–1.76)	0.001	1.41 (1.17–1.70)	0.008	1.13 (0.86–1.48)	0.378	–	–	1.53 (1.18–1.99)	0.001	1.35 (1.04–1.76)	0.026
Peritoneal involvement												
No/yes	1.62 (1.35–1.93)	<0.001	1.28 (1.07–1.53)	<0.001	1.17 (0.90–1.52)	0.248	–	–	1.89 (1.45–2.45)	<0.001	1.29 (0.56–2.98)	0.544
Perforation												
No/yes	1.21 (1.01–1.44)	0.041	1.51 (0.95–1.39)	0.149	0.95 (0.74–1.23)	0.698	–	–	1.88 (1.46–2.44)	<0.001	1.71 (1.29–2.26)	<0.001
Tumour necrosis						0.255						
Low/high	1.27 (1.07–1.50)	0.006	1.25 (1.05–1.48)	0.025	1.15 (0.90–1.47)		–	–	1.29 (0.99–1.68)	0.056	1.12 (0.85–1.46)	0.43
Mismatch repair status												
Competent/deficient	1.01 (0.81–1.25	0.94	–	–	0.96 (0.72–1.28)	0.766	–	–	1.27 (0.88–1.83)	0.203	–	–
Phenotypic subtype												
Immune	–	0.002	–	0.025	–	0.513	–	–	–	0.005	–	0.02
Canonical	1.31 (1.05–1.62)	0.015	1.19 (0.96–1.48)	0.121	0.97 (0.72–1.31)	0.841			1.80 (1.24–2.63)	0.002	1.55 (1.05–2.28)	0.029
Latent	1.32 (1.05–1.67)	0.017	1.26 (1.00–1.59)	0.051	0.94 (0.68–1.31)	0.718			1.86 (1.26–2.74)	0.002	1.75 (1.18–2.60)	0.006
Stromal	1.56 (1.23–1.99)	<0.001	1.46 (1.14–1.88)	0.003	1.26 (0.86–1.84)	0.238			1.73 (1.18–2.54)	0.005	1.75 (1.18–2.59)	0.005
Recurrence risk												
Age												
<65/<65	0.98 (0.77–1.25)	0.874	–	–	1.00 (0.67–1.49)	0.983	–	–	1.05 (0.76–1.45)	0.769	–	–
T‐Stage												
1	–	<0.001	–	0.304	–	–	–	–	–	<0.001	–	0.951
2	1.06 (0.45–2.51)	0.895	1.10 (0.44–2.74)	0.847					2.32 (0.28–19.29)	0.435	1.65 (0.19–13.96)	0.645
3	1.86 (0.87–3.98)	0.108	1.20 (0.52–2.77)	0.664					3.38 (0.47–24.33)	0.226	1.77 (0.24–13.30)	0.577
4	3.86 (1.80–8.27)	0.001	0.63 (0.22–1.79)	0.382					7.56 (1.05–54.32)	0.044	1.88 (0.21–16.73)	0.572
N‐stage												
0	–	<0.001	–	<0.001	–	–	–	–	–	0.338	–	–
1	2.73 (2.12–3.51)	<0.001	2.41 (1.86–3.12)	<0.001					2871.66 (0–high)	0.828		
2	3,51 (2.52–4.88)	<0.001	2.51 (1.77=3.56)	<0.001					3681.84 (0–high)	0.823		
Differentiation												
Poor/well or moderate	1.86 (1.35–2.56)	<0.001	1.37 (0.98–1.91)	0.067	0.97 (0.49–1.91)	0.919	–	–	1.80 (1.23–2.63)	0.002	1.67 (1.13–2.46)	0.01
Vascular invasion												
No/yes	1.97 (1.56–2.49)	0.001	1.47 (1.15–1.87)	0.002	1.38 (0.93–2.07)	0.112	–	–	1.72 (1.26–2.34)	0.001	1.49 (1.09–2.05)	0.012
Peritoneal involvement												
No/yes	2.60 (2.06–3.28)	<0.001	1.82 (1.41–2.36)	<0.001	1.92 (1.31–2.83)	0.001	1.70 (1.14–2.51)	0.008	2.46 (1.81–3.35)	<0.001	1.87 (1.33–2.62)	<0.001
Perforation												
No/yes	1.52 (1.23–1.87)	<0.001	1.38 (1.11–1.73)	0.004	1.30 (0.95–1.79)	0.106	–	–	2.01 (1.53–2.65)	<0.001	1.65 (1.22–2.23)	0.001
Tumour necrosis												
Low/high	1.19 (0.95–1.51)	0.138	–	–	0.91 (0.62–1.35)	0.654	–	–	1.26 (0.93–1.72)	0.142	–	–
Mismatch repair status												
Competent/deficient	0.81 (0.5901.12)	0.198	–	–	0.78 (0.48–1.27)	0.314	–	–	1.19 (0.77–1.84)	0.428	–	–
Phenotypic subtype												
Immune	–	<0.001	–	<0.001	–	<0.001	–	<0.001	–	0.006	–	0.011
Canonical	180 (1.30–2.50)	<0.001	1.69 (1.21–2.35)	0.002	1.03 (0.60–1.76)	0.917	1.03 (0.60–1.76)	0.916	2.20 (1.38–3.50)	0.001	2.06 (1.29–3.28)	0.002
Latent	1.98 (1.41–2.78)	<0.001	1.79 (1.27–2.53)	0.001	1.33 (0.78–2.29)	0.295	1.31 (0.77–2.26)	0.321	1.97 (1.21–3.19)	0.006	1.98 (1.22–3.22)	0.006
Stromal	3.04 (2.18–4.24)	<0.001	2.14 (1.51–3.01)	<0.001	3.07 (1.82–5.52)	<0.001	2.76 (1.63–4.68)	<0.001	2.02 (1.26–3.23)	0.003	1.98 (1.23–3.17)	0.005

**Figure 3:**
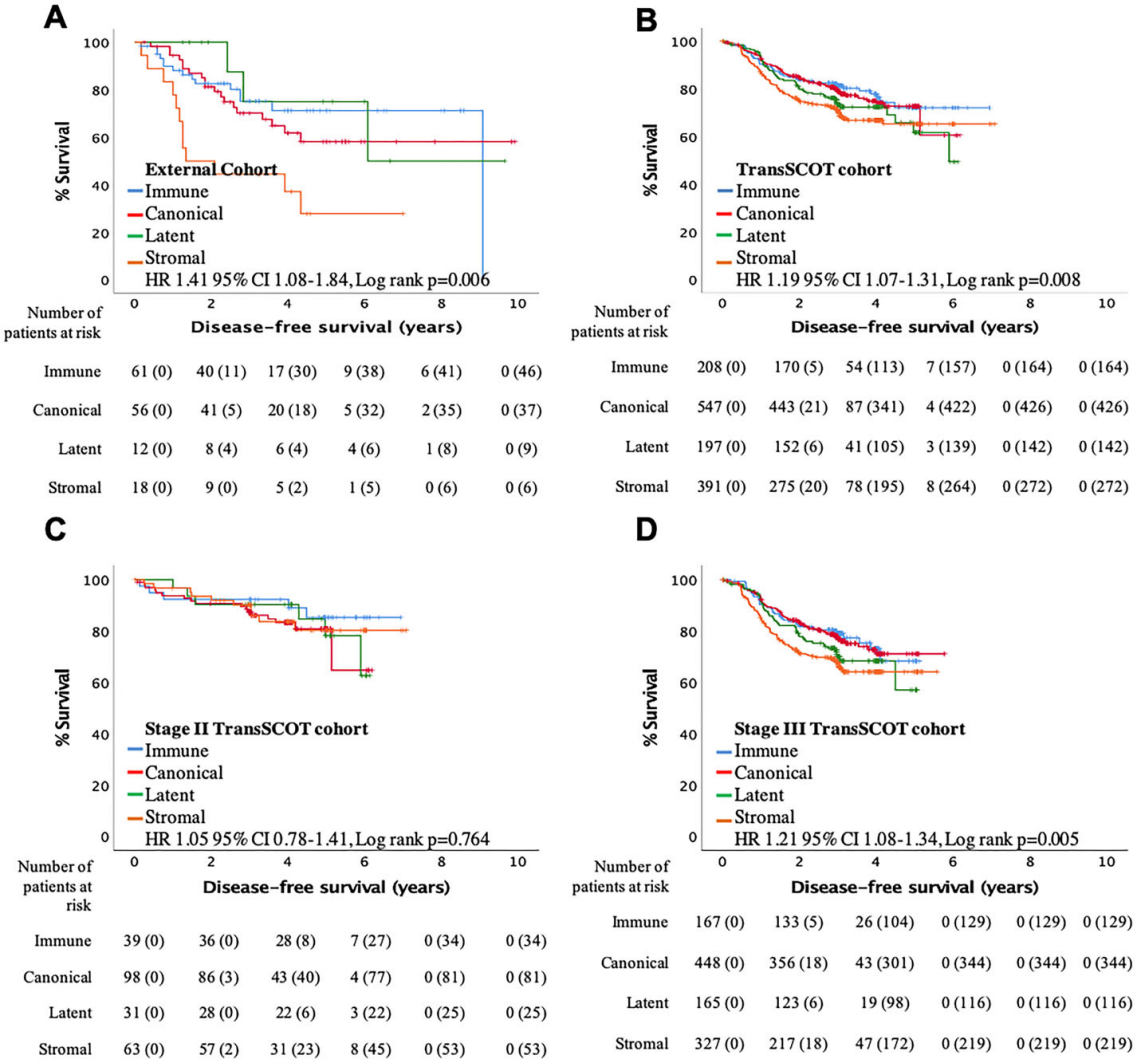
External validation confirming that phenotypic subtype stratifies disease‐free survival in patients with stage I–III CRC. (A,B) Kaplan–Meier curves comparing associations of phenotypic subtypes and disease‐free survival in (A) an external validation cohort (*n* = 146) and (B) the TransSCOT adjuvant chemotherapy cohort (*n* = 1343). (C,D) Kaplan–Meier curves comparing associations of phenotypic subtypes and disease‐free survival in (C) stage II (*n* = 231) and (D) stage III (*n* = 1107) patients from the TransSCOT adjuvant chemotherapy cohort.

External validation of the phenotypic subtypes was performed to assess the validity and translation of the subtypes within an independent laboratory. The external validation cohort contained 146 patients with the majority having stage II CRC (86%) allowing validation of the internal cohort results in an up‐to‐date early stage cohort (for cohort characteristics; see supplementary material, Table S1). Median follow‐up was 3.5 years (range 1.0–9.9 years) with 22 deaths and a 22% recurrence rate. 61 (42%) patients had an immune subtype, 55 (38%) a canonical subtype, 12 (8%) a latent subtype and 18 (12%) a stromal subtype (see supplementary material, Table S1). Phenotypic subtype significantly stratified patients by DFS (HR 1.41 95% CI 1.08–1.84, *p* = 0.006; Figure 3A), with the stromal subtype having the worst outcome. Under multivariate analysis with TNM‐stage and mismatch repair status (see supplementary material, Table S3), only phenotypic subtype (*p* = 0.028) was independently prognostic for DFS.

To validate the stage III findings in an up‐to‐date cohort, a subset of patients from the SCOT trial, the TransSCOT cohort, was utilised as it contained a high proportion of stage III patients (83%) with differing adjuvant chemotherapy regimens and durations (for cohort characteristics; see supplementary material, Table S1). All patients received FOLFOX or CAPOX adjuvant chemotherapy for at least 3 months. Median follow up was 3.0 years (range 0–7.0 years) with 339 DFS events. 208 (15%) patients had an immune subtype, 547 (41%) a canonical subtype, 197 (15%) a latent subtype and 391 (29%) a stromal subtype. To ensure this was representative of the full SCOT trial cohort, patient characteristics were compared (see supplementary material, Table S1). Both cohorts had a similar proportion of males, stage, and DFS events; the only difference was that the TranSCOT cohort had fewer rectal cancers. Therefore, the TranSCOT cohort is a reasonable representation of the overall trial population.

Phenotypic subtype significantly stratified patients by DFS (HR 1.19 95% CI 1.07–1.31, *p* = 0.008; Figure 3B), with the stromal subtype having the worst outcome. When stratified for TNM‐stage, phenotypic subtype did not associate with DFS in stage II disease (HR 1.05 95% CI 0.78–1.84, *p* = 0.522; Figure 3C) but did significantly associate with DFS in stage III disease (HR 1.21 95% CI 1.08–1.34, *p* = 0.005; Figure 3D) similar to the internal validation cohort.

**Figure 4:**
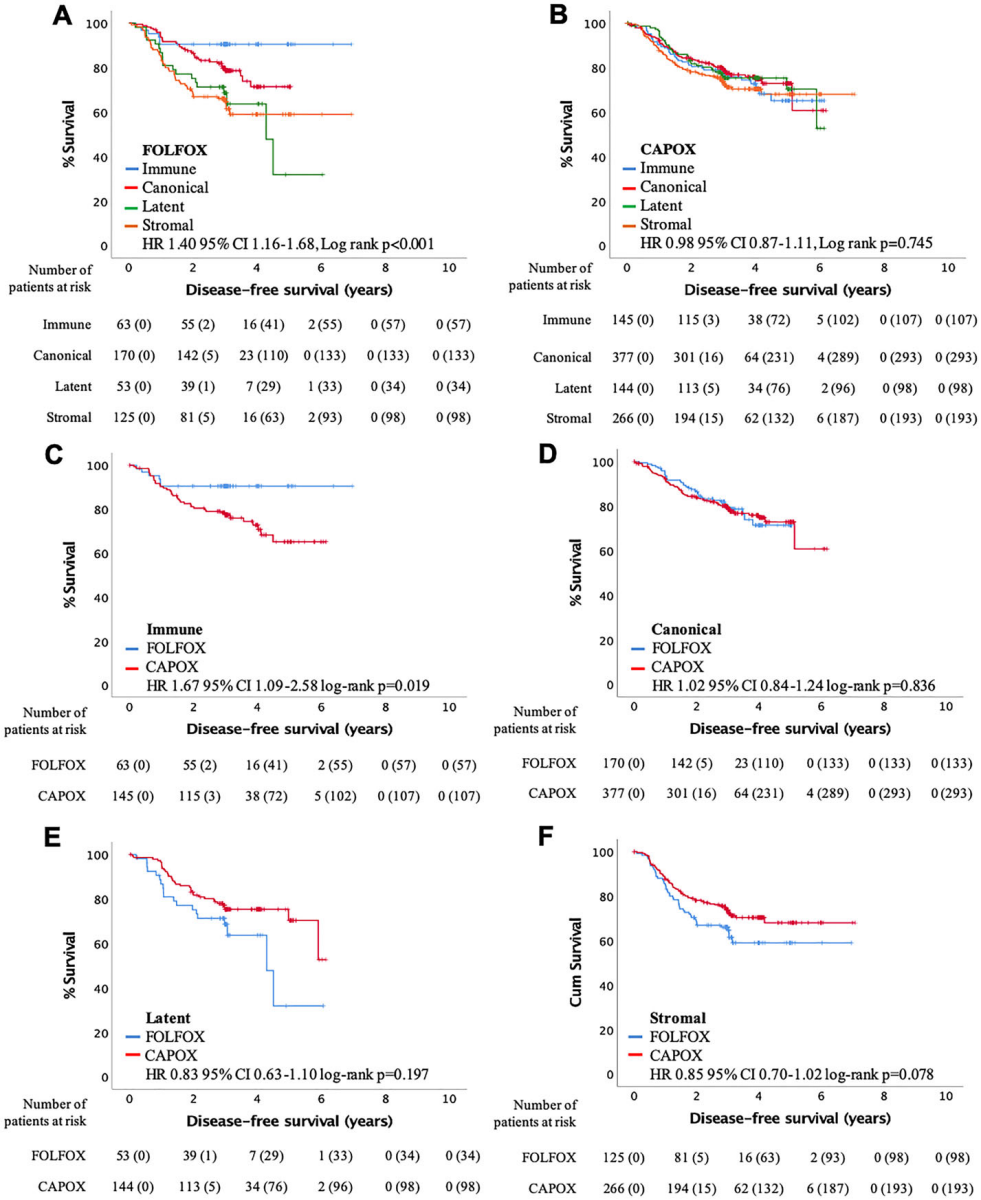
The immune subtype predicts response to FOLFOX compared to CAPOX adjuvant chemotherapy in the TransSCOT cohort (*n* = 1343). (A,B) Kaplan–Meier curves showing association of phenotypic subtype with disease‐free survival for patients receiving (A) FOLFOX (*n* = 411) or (B) CAPOX (*n* = 932) adjuvant chemotherapy within the TransSCOT cohort. (C–F)Kaplan–Meier curves showing association of chemotherapy type with disease free survival for patients with an (C) immune (*n* = 208), (D) canonical (*n* = 547), (E) latent (*n* = 197) or (F) stromal (*n* = 391) subtype.

To address the exploratory endpoint, phenotypic subtype was interrogated for associations with adjuvant chemotherapy type and duration. Multivariate cox proportional hazards analysis to assess interactions between phenotypic subtypes and chemotherapy type or duration was performed (see supplementary material, Table S4). An interaction between phenotypic subtypes and chemotherapy type (CAPOX versus FOLFOX; *p*_interaction_ = 0.011) was observed but not with duration (3 months versus 6 months; *p*_interaction_ = 0.809). As the effect of chemotherapy type depends on phenotypic subtype, associations with DFS were assessed in patients stratified for chemotherapy type (Figure 4; adjusted for T‐stage, N‐stage and treatment duration). In patients receiving FOLFOX adjuvant chemotherapy, phenotypic subtype significantly stratified DFS (HR 1.40 95% CI 1.16–1.68, *p* < 0.001; Figure 4A) with the immune subtype having the best outcome. No difference in DFS was noted in patients receiving CAPOX adjuvant chemotherapy (HR 0.98 95% CI 0.87–1.11, *p* = 0.745; Figure 4B). Furthermore, when differences in DFS were assessed between treatment types stratified by phenotypic subtype, the immune subtype showed a significant difference in DFS between the two regimens (HR 1.67 95% CI 1.09–2.58, *p* = 0.019; Figure 4C) with patients receiving FOLFOX adjuvant chemotherapy having improved outcomes. No differences in DFS between the two regimens were seen within any other phenotypic subtype (Figure 4D–F). As phenotypic subtype specifically stratified stage III patients within the full cohort, cox proportional hazards interaction analysis was repeated within stage II and stage III patients (see supplementary material, Table S4). No interactions with phenotypic subtype and treatment duration were seen for either stage. However, phenotypic subtype did interact with treatment type in stage III disease (CAPOX versus FOLFOX; *p*_interaction_ = 0.031). Therefore, patients were stratified into stage II and III disease (see supplementary material, Figure S2; adjusted for treatment duration). For patients receiving FOLFOX, DFS only associated with phenotypic subtype in stage III disease (HR 1.45 95% CI 1.20–1.76, *p* < 0.001; see supplementary material, Figure S2B). Furthermore, for patients with an immune subtype (see supplementary material, Figure S2C,D), treatment type only associated with DFS in stage III disease (HR 1.82 95% CI 1.13–2.91, *p* = 0.013; see supplementary material, Figure S2D).

Phenotypic subtype was then taken forward into multivariate analysis with clinical and therapeutic factors for DFS (Table 3). When assessing any treatment, phenotypic subtype was not independently prognostic in the full cohort (*p* = 0.374) or when restricted to only stage III patients (*p* = 0.262) compared to T‐stage and N‐stage. Phenotypic subtype was also not independently prognostic in any patient groups receiving CAPOX. However, for patients receiving FOLFOX, T‐stage (*p* = 0.018), N‐stage (*p* < 0.001) and phenotypic subtype (*p* = 0.008) were independently prognostic. Similar results were observed for stage III patients receiving FOLFOX, T‐stage (*p* = 0.046), N‐stage (*p* = 0.001) and phenotypic subtype (*p* = 0.028) were independently prognostic.

**Table 3:** Multivariate analysis of phenotypic subtypes, clinicopathological factors and disease‐free survival in the TransSCOT cohort (*n* = 1343).

	Any treatment (*n* = 1343)				FOLFOX (*n* = 411)				CAPOX (*n* = 932)			
	Univariate analysis HR (95% CI)	*P* value	Multivariate analysis HR (95% CI)	*P* value	Univariate analysis HR (95% CI)	*P* value	Multivariate analysis HR (95% CI)	*P* value	Univariate analysis HR (95% CI)	*P* value	Multivariate analysis HR (95% CI)	*P* value
All patients (*n* = 1343)												
Age												
<65/>65	0.99 (0.89–1.22)	0.917	–	–	1.14 (0.78–1.67)	0.487	–	–	0.92 (0.71–1.20)	0.541	–	–
Sex												
Female/male	1.09 (0.99–1.36)	0.414	–	–	1.16 (0.96–1.40)	0.133	–	–	1.00 (0.88–1.14)	0.975	–	–
T‐Stage												
1	–	<0.001	–	<0.001	–	0.002	–	0.018	–	<0.001	–	<0.001
2	0.79 (0.28–2.20)	0.646	0.78 (0.27–2.11)	0.63	0.31 (0.06–1.49)	0.141	0.31 (0.06–1.56)	0.156	1.52 (0.33–6.95)	0.587	1.54 (0.34–7.01)	0.58
3	1.64 (0.67–3.98)	0.279	1.56 (0.64–3.83)	0.272	0.68 (0.21–2.18)	0.518	0.56 (0.17–1.84)	0.343	3.08 (0.76–12.47)	0.115	3.29 (0.81–13.33)	0.096
4	2.94 (1.21–7.18)	0.018	2.90 (1.18–7.14)	0.012	1.31 (0.41–4.20)	0.653	0.99 (0.30–3.26)	0.984	5.39 (1.33–21.85)	0.018	6.24 (1.53–25.40)	0.011
N‐stage												
0	–	<0.001	–	<0.001	–	<0.001	–	<0.001	–	<0.001	–	<0.001
1	1.80 (1.27–2.57)	0.001	2.20 (1.54–3.15)	<0.001	1.39 (0.75–2.58)	0.297	1.52 (0.81–2.85)	0.192	2.02 (1.31–3.11)	0.001	2.58 (1.67–3.99)	<0.001
2	3.22 (2.22–4.66)	<0.001	3.47 (2.39–5.03)	<0.001	3.21 (1.71–6.00)	<0.001	3.15 (1.68–5.92)	<0.001	3.17 (2.00–5.02)	<0.001	3.51 (2.22–5.55)	<0.001
Tumour site												
Colon/rectal	0.79 (0.58–1.07)	0.123	–	–	0.77 (0.45–1.31)	0.341	–	–	0.79 (0.55–1.15)	0.222	–	–
Treatment duration												
3 months/6 months	0.98 (0.79–1.21)	0.854	–	–	0.71 (0.48–1.04)	0.074	0.72 (0.49–1.06)	0.096	1.14 (0.88–1.48)	0.322	–	–
Treatment type												
FOLFOX/CAPOX	0.96 (0.85–1.07)	0.436	–	–	–	–	–	–	–	–	–	–
Phenotypic subtype												
Immune	–	0.008	–	0.374	–	<0.001	–	0.006	–	0.525		
Canonical	1.09 (0.77–1.54)	0.621	1.03 (0.73–1.45)	0.885	2.43 (1.03–5.76)	0.044	2.26 (0.95–5.37)	0.065	0.88 (0.60–1.29)	0.512		
Latent	1.38 (0.93–2.06)	0.11	1.21(0.81–1.81)	0.343	4.43 (1.77–11.09)	0.002	3.62 (1.44–9.14)	0.006	0.96 (0.61–1.52)	0.861	–	–
Stromal	1.59 (1.13–2.25)	0.009	1.25 (0.88–1.78)	0.216	4.66 (1.99–10.92)	<0.001	3.73 (1.57–8.81)	0.003	1.12 (0.75–1.65)	0.585		
Stage III patients (*n* = 1107)												
Age												
<65/>65	0.88 (0.70–1.11)	0.281	–	–	1.01 (0.68–1.52)	0.947	–	–	0.82 (0.62–1.08)	0.165	–	–
Sex												
Female/male	1.06 (0.94–1.19)	0.35	–	–	1.17 (0.95–1.43)	0.139	–	–	1.01 (0.88–1.16)	0.867	–	–
T‐Stage												
1	–	<0.001	–	<0.001	–	0.004	–	0.046	–	<0.001	–	<0.001
2	0.69 (0.24–1.99)	0.491	0.67 (0.23–1.93)	0.456	0.21 (0.03–1.24)	0.084	0.20 (0.03–1.21)	0.08	1.43 (0.31–6.60)	0.65	1.41 (0.30–6.51)	0.662
3	1.92 (0.79–4.68)	0.15	1.71 (0.70–4.19)	0.237	0.80 (0.25–2.57)	0.706	0.59 (0.18–1.92)	0.377	3.63 (0.90–14.71)	0.071	3.41 (0.84–14–81)	0.086
4	3.59 (1.47–8.78)	0.005	3.11 (1.27–7.64)	0.013	1.47 (0.45–4.75)	0.522	0.93 (0.28–3.09)	0.905	6.83 (1.68–27.79)	0.007	6.26 (1.54–25.53)	0.001
N‐stage												
01/02	1.79 (1.42–2.25)	<0.001	1.58 (1.25–2.00)	<0.001	2.31 (1.54–3.46)	<0.001	2.07 (1.37–3.12)	0.001	1.57 (1.18–2.09)	0.002	1.36 (1.02–1.81)	0.039
Tumour site												
Colon/rectal	0.76 (0.55–1.04)	0.088	1.02 (0.74–1.42)	0.898	0.75 (0.43–1.30)	0.305	–	–	0.77 (0.52–1.13)	0.175	–	–
Treatment duration												
3 months/6 months	1.02 (0.81–1.28)	0.858	–	–	0.77 (0.52–1.16)	0.216	–	–	1.16 (0.88–1.53)	0.281	–	–
Treatment type												
FOLFOX/CAPOX	0.97 (0.86–1.10)	0.636	–	–	–	–	–	–	–	–	–	–
Phenotypic subtype												
Immune	–	0.006	–	0.262	–	0.003	–	0.028	–	0.147		
Canonical	1.04 (0.72–1.51)	0.831	0.80 (0.55–1.16)	0.239	2.88 (1.13–7.38)	0.027	2.48 (0.97–6.37)	0.059	0.77 (0.51–1.16)	0.21		
Latent	1.37 (0.90–2.09)	0.148	0.77 (0.59–1.02)	0.067	4.57 (1.69–12.39)	0.003	3.60 (1.32–9.81)	0.012	0.93 (0.57–1.51)	0.769	–	–
Stromal	1.61 (1.11–2.33)	0.012	0.95 (0.68–1.34)	0.785	4.85 (1.91–12.31)	0.001	3.67 (1.43–9.43)	0.007	1.13 (0.75–1.71)	0.56		

## Discussion

The results of the present study validate that, in the general population, histological phenotypic subtypes are an effective independent prognostic classification for patients with stage III CRC. Furthermore, phenotypic subtype can independently stratify RR across stage I–III CRC, with the stromal subtype having the highest risk of recurrence. The exploratory adjuvant chemotherapy analysis suggests that phenotypic subtype is an independent prognostic classification for stage III patients receiving FOLFOX. Interestingly, stage III patients with an immune subtype appear to respond better to FOLFOX compared to CAPOX adjuvant chemotherapy, although this exploratory analysis requires validation in an independent cohort.

Phenotypic subtype was independently associated with DFS in stage I–III CRC patients from the internal and external validation cohorts. This was attenuated in stage III patients but lost in stage II patients, suggesting this classification may have more utility in later stage disease, and may aid clinicians when assessing adjuvant therapy. Interestingly, the immune and stromal subtype showed similar prognosis at all stages, whereas prognosis was significantly worse in stage III tumours for the canonical and latent subtypes. This agrees with previous studies reporting that TSP associates with poor prognosis in CRC and other cancers independent of stage [8, 10, 11, 12]. Similarly, high intra‐tumoural lymphocytic infiltrate has previously been reported to associate with improved survival in CRC patients independent of stage [13, 14, 15, 16, 17]. However, survival effects for Ki67 proliferation rate vary in the literature suggesting that prognostic effects are dependent on other clinical factors as demonstrated by a lack of independence in multivariate analysis [18, 19]. However, this change in the prognosis of the canonical and latent subtypes from stage II (good prognosis) to stage III (poor prognosis) disease is significant for patients, as the stage II data would suggest minimal intervention. However, if the patient recurs and progresses to stage III disease it may be too late for intervention. Therefore, should these patients be put forward for adjuvant therapy for stage II disease due to their potential future prognosis if their disease recurs and progresses?

To address this further, the current study assessed RR for the phenotypic subtypes. Phenotypic subtype independently associated with risk of recurrence across all patients with stage I–III CRC, with the stromal subtype having the highest risk of recurrence and the immune subtype the lowest risk. This could be expected as TSP is known to associate with epithelial‐to‐mesenchymal transition (EMT) and invasion, which prepares the tumour for metastasis [8]. Therefore, any residual cells in stromal subtype tumours are likely to have undergone EMT and be primed for recurrence. However, similar to DFS, the canonical and latent subtypes had a low risk in stage II but a high risk in stage III disease. As both subtypes have a similar change, this suggests the effect is not dependent on proliferation rate, but potentially the low levels of immune infiltrate and stroma within the two groups. This is in line with previous findings where a low inflammatory infiltrate in stage III disease led to an unfavourable prognosis but no difference was seen between low and high immune infiltrate in stage II disease [20, 21]. Similar changes are not seen for low stroma [12], suggesting this difference may be influenced by the low immune infiltrate. This effect can be further seen in our stage II external validation cohort, where the immune, canonical and latent subtypes have a good prognosis whereas the stromal subtype has a poor prognosis similar to the internal cohort.

One difference between the results of the internal and external validation cohorts is that phenotypic subtype is an independent prognostic factor for the stage II external cohort but not for stage II patients in the internal cohort. This difference may be due to the ages of the cohorts, with the external cohort containing more recent patients with up‐to‐date adjuvant therapies. Furthermore, due to constraints on data available for the external cohort, only TNM‐stage and mismatch repair status are included in the analysis compared to multiple clinical factors for the internal cohort. Therefore, if more clinical factors were available for the external cohort this independence may be lost. Nevertheless, the external cohort validates the prognostic effect seen in the internal cohort and the translation of the method to an independent laboratory.

To assess if modern adjuvant chemotherapy regimens affect the prognostic value of the phenotypic subtypes, they were assessed in a subset of patients from the SCOT trial. In the TransSCOT cohort, phenotypic subtype could stratify DFS; however, this was not independent of other clinical factors suggesting that the adjuvant chemotherapy regimens are diminishing the prognostic effect of the subtypes. When stratified for stage, high risk stage II patients had improved DFS across all subtypes suggesting that these patients respond well to the treatment independent of subtype. Whereas, in stage III patients, with poorer DFS, a significant difference in response to chemotherapy is observed, but again this was not independent of other clinical factors.

To decipher if this loss of independent prognostic power was due to a specific treatment type or duration, interactions were investigated. A significant interaction was observed with chemotherapy type (CAPOX versus FOLFOX) but not duration (3 versus 6 months) suggesting it may be a specific type of adjuvant chemotherapy that is affecting the prognostic value of the subtypes. No survival difference was seen for patients receiving CAPOX suggesting that all subtypes had a similar response to this adjuvant therapy. However, a significant independent difference in DFS was noted for patients receiving FOLFOX, with immune patients doing significantly better compared to other subtypes, similar to stage III patients in the internal cohorts. Furthermore, when FOLFOX patients where stratified for stage, only stage III patients had an independent association with DFS, consistent with the other cohorts. This makes sense as the internal cohort patients were likely to have been treated with 5‐FU chemotherapy as used in this regimen and the external cohort was treated with a mixture of 5‐FU and FOLFOX‐based regimens. However, CAPOX utilises an oral version, capecitabine, that is metabolised to 5‐FU, so potentially this metabolic interaction may be interacting with components utilised to define the phenotypic subtypes.

To assess if the prognostic difference between the two treatment types was due to a specific subtype, patients were stratified into the four subtypes. Patients with an immune subtype did significantly better on FOLFOX compared to CAPOX especially in stage III patients, whereas patients with a stromal subtype trended towards better survival on CAPOX. Validation of these observations is required, but suggest it is the adaptive immune cells that interact differently with the two chemotherapy regimens. When immune cells are high and infiltrating the tumour as seen in the immune subtype, FOLFOX is favourable; however, when excluded, as seen in the stromal subtype, CAPOX is favourable, agreeing with previous literature reporting that patients with high tumour‐infiltrating and circulating lymphocytes had significantly improved survival when receiving FOLFOX [22, 23, 24]. One hypothesis may be that, for CAPOX, the high levels of immune cells hamper the final stage of metabolism of capecitabine inhibiting its cytotoxic effect. If this observation is validated, the immune subtype is a promising marker to identify patients more likely to benefit from FOLFOX rather than CAPOX adjuvant chemotherapy.

This is the first histological CRC subtyping method to have independent prognostic power in stage III patients as well as associations with risk of recurrence and adjuvant chemotherapy. The method also utilises sections routinely prepared within clinical pathology laboratories making it easy to translate to clinical practice. As well as being readily translated to clinical practice and being cost effective, phenotypic subtype can also classify all patient tumours, unlike the CMS classification where 20% of tumours are unclassifiable [1]. Phenotypic subtype assesses cell types within the tumour and microenvironment separately whereas the CMS classification looks at all cell types together. In contrast, the cancer‐cell intrinsic subtypes only utilise tumour‐cell specific transcriptomic analysis, which the author suggests alleviates any issues with tumour heterogeneity [2]; however, this ignores the tumour microenvironment known to be vital for both anti‐tumour and pro‐tumour mechanisms. The phenotypic subtypes take all of this into account in a simple, readily translated and cost‐effective way utilising routine clinical methods, unlike the above transcriptomic‐based classifiers, which have failed to be translated into clinical practice due to issues with robustness/reproducibility, turnaround time, and high associated costs.

In conclusion, in the general population, the histological phenotypic subtype classification has independent prognostic power for patients with stage III CRC. Furthermore, phenotypic subtype can predict risk of recurrence for these patients, with the immune subtype having a significantly diminished risk compared to the other three subtypes. In an adjuvant chemotherapy trial population, phenotypic subtype may independently predict response to FOLFOX adjuvant chemotherapy within stage III patients, with the immune subtype having a better response to this treatment when compared to CAPOX. Going forward, the utility of these subtypes will be distinguishing the optimal treatments for each group, including established therapeutics or the development of novel interventions. Another important area of research is to develop a digital pathology approach to distinguish the phenotypic subtypes utilising deep learning to allow a quick automated analysis of the patient tissue, either by automating and integrating the current manual approaches or developing surrogate markers utilising neural classification networks. Upon further validation, the histological phenotypic subtype classification could be a useful aid in the clinic for CRC prognosis, particularly for stage III disease, identifying patients with a risk of recurrence and patients who could benefit from FOLFOX adjuvant chemotherapy.

## Author contributions statement

AKR, JHP, CSDR, PH, DCM, OS and JE designed the experiments. StH, AGMTP, ER, KS, LV, AH, JG, DNC, IT, MS, TJI and JE developed the cohorts. AKR, JHP and AH carried out the study. AKR and JP analysed the data. AKR wrote the manuscript. All authors reviewed the manuscript and approved the final version for submission and publication.

## Supporting information


**Figure S1.** Proportion of common clinical characteristics between each phenotypic subtype
**Figure S2.** Response to adjuvant chemotherapy in stage II versus stage III patients from the TransSCOT cohort
**Table S1.** Patient characteristics for cohorts
**Table S2.** Multivariate analysis for components of the phenotypic subtype classification for DFS and recurrence risk
**Table S3.** Multivariate analysis of phenotypic subtypes, clinicopathological factors and disease‐free survival in the external validation cohort
**Table S4.** Multivariate interaction analysis of phenotypic subtypes, chemotherapy type and chemotherapy duration in the TransSCOT adjuvant chemotherapy cohort
