# Supplementary material for: Histological phenotypic subtypes predict recurrence risk and response to adjuvant chemotherapy in patients with stage III colorectal cancer
Source: J Pathol Clin Res. 2020 May 13;6(4):283–96. doi: 10.1002/cjp2.171 (PMC7578335; doi:10.1002/cjp2.171)
Supplement: Supplementary file 1 — Figure S1. Proportion of common clinical characteristics between each phenotypic subtype Figure S2. Response to adjuvant chemotherapy in stage II versus stage III patients from the TransSCOT cohort Table S1. Patient characteristics for cohorts Table S2. Multivariate analysis for components of the phenotypic subtype classification for DFS and recurrence risk Table S3. Multivariate analysis of phenotypic subtypes, clinicopathological factors and disease‐free survival in the external validation cohort Table S4. Multivariate interaction analysis of phenotypic subtypes, chemotherapy type and chemotherapy duration in the TransSCOT adjuvant chemotherapy cohort [file CJP2-6-283-s001.docx]

**Histological phenotypic subtypes predict recurrence risk and response to adjuvant chemotherapy in patients with stage III colorectal cancer**

AK Roseweir *et al*. *J Pathol Clin Res* DOI: 10.1002/cjp2.171

**Supplementary Material**


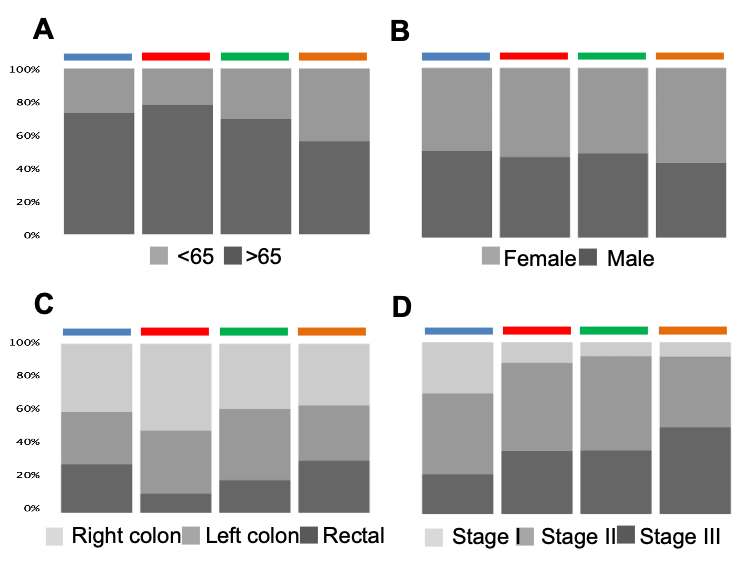


**Figure S1. Proportion of common clinical characteristics between each phenotypic subtype.** (A-D) Histograms showing proportions of (A) age, (B) sex, (C) tumour location and (D) TNM-stage within each phenotypic subtype for patients within the internal cohort.

**Figure S2. Response to adjuvant chemotherapy in stage II versus stage III patients from the TransSCOT cohort.** (A, B) Kaplan Meier curves showing association of phenotypic subtype with disease-free survival in patients receiving FOLFOX adjuvant chemotherapy with (A) stage II (n=66) or (B) stage III (n=344) disease. (C, D) Kaplan Meier curves showing association of chemotherapy type with disease free survival for patients with an immune subtype and (C) stage II (n=39) or (D) stage III disease (n=167).

**Table S1. Patient characteristics for cohorts**

|  | **Internal Cohort**  **n=893 (%)** | **External Validation cohort n=146 (%)** | **TransSCOT**  **Cohort**  **n=1343 (%)** | **Full SCOT trial Cohort**  **n=6088 (%)** |
| --- | --- | --- | --- | --- |
| **Age**  **<65**  **>65**  **Unknown** | 280 (31)  613 (69)  0 (0) | 29 (20)  52 (36)  65 (44) | 727 (54)  616 (46)  0 (0) | -  -  - |
| **Sex**  **Female**  **Male**  **Unknown** | 431 (48)  462 (52)  0 (0) | 44 (30)  37 (25)  65 (45) | 536 (40)  805 (60)  0 (0) | 2401 (39)  3687 (61)  0 (0) |
| **TNM-stage**  **I**  **II**  **III** | 127 (14)  437 (49)  329 (37) | 2 (2)  126 (86)  18 (12) | 5 (0)  231 (17)  1107 (83) | 20 (0)  1096 (18)  4972 (82) |
| **Tumour Location**  **Colon**  **Rectal**  **Unknown** | 667 (75)  226 (25)  (0) | 134 (92)  12 (8)  0 (0) | 1116 (83)  227 (17)  0 (0) | 4334 (71)  1754 (29)  0 (0) |
| **Adjuvant Therapy**  **No**  **Yes**  **Unknown** | -  -  - | 37 (25)  20 (14)  89 (61) | 0 (0)  1343 (100)  0 (0) | (0)  6088 (100)  0 (0) |
| **MMR Status**  **Competent**  **Deficient**  **Unknown** | 711 (80)  161 (18)  21 (2) | 107 (73)  37 (25)  2 (2) | -  -  - | -  -  - |
| **Phenotypic Subtypes**  **Immune**  **Canonical**  **Latent**  **Stromal** | 305 (34)  248 (28)  186 (21)  154 (17) | 61 (42)  55 (38)  12 (8)  18 (12) | 208 (15)  547 (41)  197 (15)  391 (29) | -  -  -  - |
| **Recurrence**  **No**  **Yes**  **Unknown** | 601 (67)  292 (33)  0 (0) | 114 (78)  32 (22)  0 (0) | 1004 (75)  339 (25)*  0 (0) | 4441 (73)  1624 (27)*  0 (0) |
| **Survival**  **Alive**  **Dead**  **Cancer Death**  **Non-cancer Death**  **Unknown** | 335 (37)  558 (63)  256 (29)  287 (32)  15 (2) | 99 (68)  37 (32)  -  -  0 (0) | 1004 (75)  339 (25)*  -  -  0 (0) | 4441 (73)  1624 (27)*  -  -  23 (0) |

*events being recurrence or death (separate data not available)

**Table S2: Multivariate analysis for components of the phenotypic subtype classification for DFS and recurrence risk (n=893)**

|  | **Univariate analysis**  **HR (95% CI)** | **P-value** | **Multivariate analysis**  **HR (95% CI)** | **P-value** |
| --- | --- | --- | --- | --- |
| **Disease-free survival (n=881)** |  |  |  |  |
| **Klintrup Makinen grade**  weak/strong | 0.73 (0.61-0.87) | 0.001 | 0.75 (0.62-0.89) | 0.002 |
| **Tumour Stroma percentage**  <50%/>50% | 1.24 (1.02-1.50) | 0.030 | 1.19 (0.98-1.44) | 0.073 |
| **Ki67 proliferation index**  <30%/>30% | 0.96 (0.81-1.13) | 0.613 | 1.00 (0.85-1.19) | 0.971 |

**Table S3. Multivariate analysis of phenotypic subtypes, clinicopathological factors and disease-free survival in the external validation cohort (n=146)**

|  | **Univariate analysis**  **HR (95% CI)** | | **P-value** | **Multivariate analysis**  **HR (95% CI)** | **P-value** |
| --- | --- | --- | --- | --- | --- |
| **TNM Stage**  I  II  III | | -  0.33 (0.05-2.46)  0.68 (0.09-5.40) | 0.087  0.282  0.716 | -  0.48 (006-3.61)  0.89 (0.11-7.18) | 0.195  0.475  0.913 |
| **Mismatch Repair Status**  competent/deficient | | 0.845 (0.43-1.65) | 0.623 | - | - |
| **Phenotypic Subtype**  immune  canonical  latent  stromal | | -  1.34 (0.68-2.64)  0.75 (0.22-2.60)  3.43 (1.60-7.35) | 0.007  0.404  0.649  0.002 | -  1.28 (0.65-2.53)  0.76 (0.22-2.62)  2.92 (1.35-6.34) | **0.028**  0.474  0.658  0.007 |

**Table S4. Multivariate interaction analysis of phenotypic subtypes, chemotherapy type and chemotherapy duration in the TransSCOT adjuvant chemotherapy cohort (n=1343)**

|  | **Immune (n)** | **Canonical (n)** | **Latent (n)** | **Stromal (n)** | **Interaction p-value** |
| --- | --- | --- | --- | --- | --- |
| **All patients (n=1343)** | | | | | |
| **Chemotherapy Type** | 208 | 547 | 197 | 391 | 0.011 |
| **Chemotherapy duration** | 208 | 547 | 197 | 391 | 0.809 |
| **Stage II patients (n=231)** |  |  |  |  |  |
| **Chemotherapy Type** | 39 | 98 | 31 | 63 | 0.070 |
| **Chemotherapy duration** | 39 | 98 | 31 | 63 | 0.982 |
| **Stage III patients (n=1107)** | | | | | |
| **Chemotherapy Type** | 167 | 448 | 165 | 327 | 0.031 |
| **Chemotherapy duration** | 167 | 448 | 165 | 327 | 0.923 |
